# Whey as an Environmental Issue and Its Possible Solutions: Its Utilization as Culture Medium to Produce L‐Threonine Through *E. coli* in a Bioreactor

**DOI:** 10.1155/bri/3996383

**Published:** 2026-01-23

**Authors:** Sara Pineda Vélez, Yudy Natalia Cortés Velásquez, Claudia Patricia Sánchez Henao, Jhon Fredy Vélez Blandón

**Affiliations:** ^1^ Nutrition and Food Technology Research Incubator, Pharmaceutical and Food Sciences Faculty, Universidad de Antioquia, Medellín, 50010, Colombia, udea.edu.co; ^2^ Nutrition and Food Technology Research Group, Pharmaceutical and Food Sciences Faculty, Universidad de Antioquia, Medellín, 50010, Colombia, udea.edu.co

**Keywords:** biorefinery, culture medium, downstream processing, economic viability, essential amino acids, functional molecule, metabolic engineering, nutritional supplement, sustainability, waste valorization

## Abstract

Whey, a by‐product of the cheese manufacturing industry, represents one of the most abundant and polluting effluents in the global food industry. Despite traditionally being underutilized and often discarded, its rich nutrient profile, particularly protein and lactose, has increasingly sparked an interest in its value within biotechnological processes. This review analyses the potential of whey as a sustainable substrate for the microbial production of value‐added bioproducts, focussing on L‐threonine production as a strategic case study, while addressing the environmental impact of inadequate disposal and current utilization strategies. A comparative analysis with other agroindustrial waste demonstrates whey’s competitive advantages in terms of composition, cost‐effectiveness and sustainability metrics. Furthermore, L‐threonine biological and industrial importance, and the most relevant advances in metabolic engineering, optimized fermentation and emerging tools such as optogenetics and machine learning are discussed, as they facilitate enhanced L‐threonine yields through the creation of robust, high‐producing strains. Technoeconomic analysis at pilot scale (33.8 tons/year) indicates that whey‐based production offers a comparative cost advantage of 7.4% over glucose‐based processes (20.55 USD/kg vs. 22.20 USD/kg). While absolute costs at pilot scale exceed current industrial market prices (1.31–1.66 USD/kg)—reflecting typical scale effects—the demonstrated comparative advantage and substantial environmental benefits (waste valorization, elimination of disposal costs and circular economy alignment) position whey‐based L‐threonine production as a strategic biorefinery opportunity with significant potential for industrial‐scale implementation. This cost benefit is primarily driven by the lower market price of whey compared to commercial glucose substrates, which compensates for the slightly higher downstream processing costs (5.90 vs. 5.40 USD/kg) required for complex matrices. Downstream processing considerations, including recovery, purity requirements and economic viability, are comprehensively addressed. This review concludes that whey, far from being merely a pollutant, has the characteristics required to become an asset for biotechnology. Utilizing whey as a culture medium for L‐threonine production by *E. coli* in bioreactors not only offers a solution to mitigate a significant environmental issue but also opens a path for the cost‐effective, sustainable production of a globally high‐demand amino acid. Whey represents a strategic biorefinery platform with potential for industrial‐scale implementation. Continued research and development in this area are fundamental to fully realizing this potential.

## 1. Introduction

Agroindustrial activity has grown and developed in response to the increase in the world’s population. As a result, a large quantity and variety of wastes are released daily into the air, water sources and ground, largely without proper treatment, thus creating environmental problems that threaten human health due to the overall deterioration of the environment’s quality. Agroindustrial waste includes cereal straw (wheat, rice) [1], grain husks and bran (barley, rice, wheat) [2, 3], sugarcane residues (bagasse, vinasses) [4, 5], oil palm residues (empty bunches, leaves, fibres, kernels) [6] and coffee residues (husk, pulp) [7], among others.

Increased awareness of environmental conservation has driven the study and development of options that allow for the use and valorization of agroindustrial wastes [7, 8], contributing to reducing their negative environmental impact. In relation to the waste generated in coffee production, the use of coffee grounds as a substrate for ethanol production using the *Saccharomyces cerevisiae* KTCT7226 strain has been reported [9], as well as in the production of enzymes (beta (β)‐mannanase using a recombinant *Aspergillus sojae* AsT3 and inulinase using *Aspergillus niger* A42) and oligosaccharides (mannooligosaccharides, fructooligosaccharides) by solid‐state fermentation [10]. Coffee pulp has been used as a substrate in hydrogen production using mutated strains of *Escherichia coli* BW25113 [11], in the production of α‐amylase in solid‐state fermentation using a fungal strain of *Neurospora crassa* CFR 308 [12] and in the production of polygalacturonase by solid‐state fermentation of an *Aspergillus niger* strain called van Tieghem [13]. No reports were found in the open literature on the use of coffee waste as a substrate in the microbiological production of amino acids.

Wastes derived from the production of refined sugar from sugarcane have also been reported to be used in the microbial production of valuable metabolites. For example, vinasse and bagasse were used for hydrogen production by anaerobic digestion [14]. The bioconversion of sugarcane molasses waste to high‐value exopolysaccharides by engineered *Bacillus licheniformis* has also been reported [15]. The use of cane bagasse in the production of pectinases by solid‐state fermentation with *Aspergillus oryzae* [16] and in the production of cellulases and xylanases in solid‐state fermentation by the strains *Aspergillus niger* CECT 2700, CECT 2915 and ITV‐01 [17], in bioethanol production through simultaneous saccharification and fermentation by *Saccharomyces cerevisiae* BY4743 [18] and in the production of L‐tyrosine by *Escherichia coli* (*Ec*) strain Tyr01 modified to overexpress two key genes for L‐tyrosine production (*tyrAfbr* and *aroGfbr*) [19].

On the other hand, residues generated in palm oil production have been studied to produce flocculant via solid‐state fermentation process using oil palm empty fruit bunch fibres as substrate by *Aspergillus niger* DWB [20]. Palm fibres have been used as a phase for the adsorption of invertase in the production of inverted sugars by Baker’s yeast [21]. Palm oil mill effluent wastes and palm oil decantation cakes were used as substrates to produce antimicrobial agents using fermentation by *Streptomyces philanthi* RM‐1‐138 [22]. Palm oil mill effluent and empty fruit bunch wastes were used as substrates in a cofermentation process involving *Lysinibacillus* sp. and *Aspergillus flavus* for fuel production [23]. At the time of submitting this article, no reports were found on the use of palm oil production wastes in microbial amino acid production.

Another agroindustrial waste that has been shown to represent a serious risk to the environment is whey, which is the main waste from the dairy industry and significantly affects both water sources and the soil where it is discharged [24]. Due to its high content of carbohydrates (D‐lactose) and other organic compounds, it has demonstrated its potential in a variety of applications, for example, for the microbiological production of amino acids, including essential amino acids such as L‐threonine (Thr) [25], a key amino acid for the proper functioning of organs and tissues in humans. This review aims to highlight whey as a potential substrate in the microbiological production of amino acids, especially Thr.

## 2. Whey Production

Whey, or dairy whey, is a waste that comes from the cheese manufacturing process. This process consists of adding an enzyme or acid to the milk, which causes most of the protein content to clot. The result is a semisolid product rich in casein and fat that, after ripening and drying, becomes cheese. When the solid is removed, dairy whey is obtained: a green‐yellowish and slightly acidic‐flavoured liquid [27].

It is estimated that, worldwide, between 190 and 200 million tons of whey are produced every year as a result of large‐scale dairy product manufacture [35]. According to the latest data reported, approximately 0.623 million tons of whey were produced in Colombia in 2022 [36]. This by‐product is one of the most abundant in the food industry, and its production volume is directly related to cheese and other dairy product manufacture. Particularly, to make 1 kg of cheese, between 10 and 12 L of milk are required. From this process, about 8–9 L of whey is generated, which demonstrates the significant volume of the waste produced [37].

Processing a portion of whey currently allows for its valorization and obtaining products with major functional and commercial interest. Its content, rich in nutrients and versatility, explains its increasingly utilization in different applications in the food, pharmaceutical and biotechnological sectors. This product is used in the manufacture of fermented, alcoholic and refreshing beverages, as well as in the production of protein concentrates, hydrolysates and isolates. It is also used in bakery products, processed cheese and child formula [26]. In biotechnology, it works as a substrate to produce ethanol, organic acids (such as lactic and citric), microbial biomass, exopolysaccharides such as xanthan gum and biodegradable biopolymers with applications in food packaging [26]. On the other hand, in the agricultural sector, whey has been traditionally used for animal feeding and as a fertilizer. However, its most valuable use is related to protein and its technological functionalities because of its gelling, foaming, binding, emulsifying and stabilizing properties, which allow its inclusion in a wide array of food and pharmaceutical products (see Table 1) [31].

**Table 1:** Overview of whey utilization in different industrial sectors (biotechnology, agriculture, pharmaceutical and food), including their specific uses and the resulting products.

Sector	Application	Description/products obtained	Reference
Biotechnology	• Substrate for ethanol production (culture medium)	Microbial fermentation to obtain ethanol as biofuel	[26]
	• Substrate in organic acid production (lactic, citric)	Used in food, pharmaceuticals and biodegradable plastics	[27]
	• Substrate in microbial biomass production	Culture of microorganisms for unicellular or probiotic proteins	[28–30]
	• Substrate in exopolysaccharides synthesis (xanthan gum)	Emulsifying and thickening agents in foods and cosmetics	[31]
	• Substrate in biodegradable biopolymer production	Sustainable food packaging	[32]

Food	• Protein supplements	Shakes, protein bars	[33]
	• Functional ingredient	Gelling, foaming, emulsifying agents (i.e., in yoghurts, bakery, supplements)	

Agricultural	• Organic fertilizer	Contribution of nitrogen and other nutrients to the soil.	[34]
	• Animal feed	Porcine feed.	

Pharmaceutical	• Functional ingredient.	Encapsulation of pharmaceuticals or protein supplements.	[33]

## 3. Chemical Composition and Environmental Impact

Chemical composition of whey changes depending on its origin and processing, thus being divided into two main types: sweet whey, with a pH between 5.8 and 6.6, produced by using enzymes like rennin, and acidic whey, whose pH is below 5, produced by clotting with organic or mineral acids [38]. Their composition profiles differ significantly, as shown in Table 2.

**Table 2:** Compositional characterization of sweet and acid whey, showing the percentage of the main components present in each type, including predominant nutrients and total solid contents.

Component	Sweet whey (g/L)	Sweet whey (%)^∗^	Acid whey (g/L)	Acid whey (%)^∗^
Total solids	63.0–70.0	6.28–6.98	63.0–70.0	2.28–6.98
Lactose	46.0–52.0	4.59–5.19	44.0–46.0	4.39–4.59
Proteins	6.0–10.0	0.60–1.00	6.0–8.0	0.60–0.80
Calcium	0.4–0.6	0.04–0.06	6.0–8.0	0.60–0.80
Phosphates	1.0–3.0	0.10–0.30	2.0–4.5	0.20–0.45
Lactate	2.0	0.20	6.4	0.64
Chlorides	1.1	0.11	1.1	0.11

*Note:* Data taken from [39].

^∗^Calculated using the density reported by [40].

This by‐product is a rich source of protein, with 0.8% and 1.5% of its composition. Among the most prominent are α‐lactalbumin, β‐lactoglobulin and bovine serum albumin [38]. Regarding carbohydrate content, lactose is the main component, with a concentration between 4.04% and 4.55%. This compound not only influences the organoleptic properties of whey but also improves water retention capacities and contributes to the texture and creaminess of derivative products [26]. Lipids are present in a lesser proportion (0.09%–0.29%). From those, approximately 30% correspond to nonsaturated fatty acids that are beneficial for health. Furthermore, it is a source of minerals such as calcium, phosphorus, potassium and magnesium, representing between 0.6% and 0.78% of its composition [26].

However, even though whey has started to increase value in sectors such as the food and pharmaceutical industries, a considerable part of this by‐product is not properly utilized yet. Therefore, it is disposed of into rivers or soil without any treatment, thus representing an environmental issue. As stated earlier, whey has a high organic load that significantly increases contamination levels wherever it is disposed of. When discharged into soils, it can cause alterations in their physicochemical structure, reducing fertility and affecting agricultural yield. In water, the presence of whey reduces levels of dissolved oxygen, which causes a negative impact on aquatic biodiversity. It is estimated that, for every 100 kg of liquid whey, approximately 3.5 kg of biological oxygen demand (BOD) and 6.8 kg of chemical oxygen demand (COD) are produced, which represents a high polluting load for the water sources where the whey is poured [41]. Lactose is the main component responsible for this high pollutant load [26].

Given this complex composition and its highly contaminating potential when not properly managed, there is a need to explore innovative strategies for its utilization. In this context, fermentative processes emerge as a promising alternative to transform this waste into products with a high added value.

## 4. Whey in Bioprocesses

At the research level, whey has proven to be a valuable substrate in fermentative processes due to its low cost, availability and nutritional content. Its use as a carbon source has been effective in essential amino acid production through microbial fermentation. In the case of L‐valine, studies with genetically modified *Ec* evinced an increase of 36% in yield when using whey versus glucose or lactose because of its association with a higher cellular growth and amino acid synthesis [42]. Likewise, production of L‐threonine using *Ec ATCC 21277*, combining whey with tilapia entrails hydrolysate, has been reported. The process, experimentally optimized, reached yields of up to 0.41 g/L, which confirms technical feasibility and the potential of whey in sustainable fermentative processes [25].

### 4.1. Comparative Advantages of Whey Versus Other Agroindustrial Substrates

The selection of an appropriate substrate is crucial for the economic and technical viability of microbial fermentation processes. While various agroindustrial wastes have been explored as culture media for amino acid production, they present distinctive advantages that position it as a strategic substrate. This section provides a comprehensive comparative analysis of whey against other commonly used substrates, including molasses, corn steep liquor (CSL) and lignocellulosic hydrolysates, considering technical, economic and sustainability aspects.

From a compositional perspective, whey offers a balanced nutrient profile particularly suitable for microbial growth and metabolic activity. Unlike molasses, which is primarily composed of sucrose (45%–55%) with limited nitrogen content (1%–2%), they provide both readily available lactose (4.5%–5%) and high‐quality protein (0.8%–1.5%), reducing or eliminating the need for expensive nitrogen supplements such as yeast extract or peptone. This represents a significant cost advantage, as nitrogen supplementation can account for 30%–40% of total medium costs in industrial fermentation [43]. CSL, while rich in nitrogen and amino acids, requires careful pH management and often contains inhibitory compounds that can reduce microbial productivity. Lignocellulosic hydrolysates, despite being abundant and low‐cost, present major technical challenges including the presence of fermentation inhibitors (furfural, hydroxymethylfurfural, phenolic compounds) that require extensive pretreatment and detoxification steps, significantly increasing processing costs and complexity [32].

Economic analysis reveals compelling advantages for whey‐based fermentation. Based on detailed technoeconomic calculations using concentrated lactose from whey, the substrate cost for Thr production is significantly lower: whey‐based medium at approximately 0.027 USD/L compared to glucose‐based medium at 0.092 USD/L, representing a 71% reduction in medium costs. Market prices for substrates show concentrated lactose from whey at 0.20 USD/kg, molasses at 0.20–0.35 USD/kg, CSL at 0.30–0.50 USD/kg and glucose at 3.46 USD/kg. Additionally, whey often has a negative value for dairy producers facing disposal costs, creating opportunities for mutually beneficial arrangements. Complete production cost analysis (including raw materials, operational costs and downstream processing [DSP]) demonstrates that whey‐based Thr production achieves 20.55 USD/kg compared to 22.20 USD/kg for glucose‐based production, representing a 7.4% overall cost advantage [39, 43–45].

Pretreatment requirements constitute another critical differentiating factor. Whey typically requires minimal pretreatment: basic filtration to remove suspended solids, pH adjustment and thermal sterilization. In contrast, lignocellulosic biomass demands complex pretreatment involving acid/alkaline hydrolysis, enzymatic saccharification and detoxification, processes that can consume 40%–60% of the total production cost [43]. Molasses requires dilution, pH adjustment and clarification to remove suspended materials and reduce viscosity, while CSL needs careful standardization to ensure batch‐to‐batch consistency. The relative simplicity of whey preprocessing reduces capital investment requirements and operational complexity, facilitating technology transfer and scale‐up [46].

From a sustainability perspective, whey valorization offers multiple environmental benefits beyond substrate provision. The dairy industry generates approximately 190–200 million tons of whey annually, with significant portions inadequately managed, leading to environmental pollution. Utilizing whey as a fermentation substrate simultaneously addresses waste management challenges and reduces demand for purpose‐grown feedstocks, contributing to circular economic principles. Life cycle assessment studies indicate that whey‐based amino acid production can reduce carbon footprint by 35%–45% compared to glucose‐based processes, primarily due to avoided whey disposal emissions and reduced agricultural inputs [35]. Furthermore, its utilization can generate carbon credits under certain regulatory frameworks, providing additional economic value. Table 3 presents a comprehensive comparison of key technical and economic parameters for different substrates used in L‐threonine fermentation, highlighting the competitive positioning of whey in multiple dimensions.

**Table 3:** Comparative analysis of agroindustrial substrates for Thr production.

Substrate	Carbon content (%)	Nitrogen content (%)	Pretreatment complexity	Cost (USD/kg)	Thr yield (g/L)	Reference
Whey	4.50–5.00 (lactose)	0.80–1.50 (protein)	Low	0.05–0.15	0.35–0.45	[25]
Molasses	45–55 (sucrose)	1–2	Medium	0.20–0.35	0.40–0.55	[48]
Corn steep liquor	15–20	4–6	Medium	0.30–0.50	0.45–0.60	[49]
Lignocellulosic	30–40 (post‐treatment)	< 0.50	Very high	0.40–0.80	0.25–0.40	[32]
Glucose (pure)	100	0	None	0.30–0.50	0.50–0.70	[50]

However, it is important to acknowledge certain limitations of whey as a fermentation substrate. The variable composition depending on cheese type and production process requires robust process control and potential medium standardization. The presence of residual fats and proteins, while nutritionally beneficial, can complicate downstream purification of the target product, potentially increasing recovery costs by 15%–25% compared to pure glucose‐based media. Additionally, lactose utilization requires appropriate microbial strains with efficient lactose metabolism, which may necessitate metabolic engineering efforts for organisms naturally preferring glucose [47].

Despite these challenges, the overall technoeconomic analysis strongly favours whey as a preferred substrate for L‐threonine and other amino acid production, particularly when considering the dual benefits of waste valorization and sustainable bioprocessing. The continued development of robust fermentation protocols, strain optimization for lactose utilization, and efficient downstream processing methods will further strengthen whey’s competitive position in the growing amino acid market.

## 5. Relevance of L‐Threonine

Amino acids are named after the presence of a carboxylic acid group and an amino group in their structure. They are biomolecules that compose proteins due to the formation of peptide bonds between amino acid molecules and, consequently, every living tissue and organism. They are so important that it is common to call them *life builders.* In nature, around 300 amino acids have been discovered. Nonetheless, human beings require only 20 of them; these have been classified from a nutritional approach with a direct focus on growth and the ability to be synthesized or not autonomously. This ability created the essential group, which includes L‐histidine, L‐isoleucine, L‐leucine, L‐lysine, L‐methionine, L‐phenylalanine, Thr, L‐tryptophan and L‐valine; all of them are not synthesized by the organism, which means they must be obtained through diet [51].

The concept of essential and nonessential amino acids has been constantly researched and discussed for the last 40 years. From this discussion has come the term “conditionally essential amino acid,” which refers to those whose synthesis may be possible but can be limited by factors such as inadequate supplementation of their precursors, maturity and the health status of the individual. A common example is tyrosine, synthesized from phenylalanine through the hydroxylase phenylalanine enzyme, whose presence is not common in babies and small children, thus making tyrosine an amino acid they must also obtain from their diet [52].

Following the functional approach, Thr is one of the amino acids that have increased its relevance not only as necessary for protein construction, but also as a key element for intestinal health and immunological response. Studies have demonstrated its importance in intestinal mucin synthesis, modulation of immune responses and cellular differentiation. Between 40% and 60% of dietary Thr is captured and used at the intestinal level, prioritized for mucosal synthesis, where approximately 30% of mucin total amino acids is Thr. Consequently, supplementation with Thr has risen as a promising means to reduce antibiotic use in animal production, and its deficiency has been associated with hepatic triglyceride accumulation, intestinal disease and impaired muscle growth [53–56].

There are not many studies carried out in humans, but their results raise hopes given that Thr supplementation significantly reduced spasticity signs [57], and its presence in mother’s milk was correlated with intestinal immunomodulation of lactating children to members of the *Enterobacteriaceae* family [58]. This way, with scientific evidence, it was determined that the recommended Thr daily dose for healthy adults set by the FDA in 1985 of 7 mg K^−1^ had to be increased to 15 mg K^−1^ [59].

Beyond nutritional applications, Thr shows promising potential in nanotechnology and pharmaceutical delivery systems. It has been identified as a precursor for nickel nanoparticles useful in microwave‐absorbing materials and battery manufacturing [60]. Recent breakthroughs include its use as a precursor of Thr polyurethane, a particle that performs as a medication liberation agent with high drug encapsulation efficiency (60%–90%) [61], and as a suitable precursor for the production of L‐2‐aminobutyrate, an important chiral intermediate drug in modern medicine [62]. These diverse applications across nutritional, pharmaceutical and industrial sectors underscore the strategic value of developing cost‐effective production methods using sustainable substrates such as whey.

## 6. L‐Threonine Microbial Production

**Figure 1:**
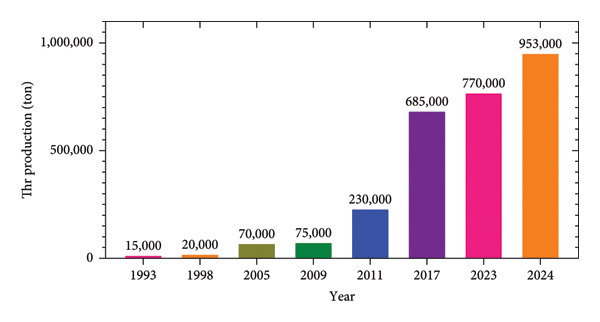
Thr world production. Created from data published by [63, 65, 66, 68–72].

Thr is a highly biologically important amino acid for mammals, a reason that provides it with several applications at the industrial level that generates a demand of approximately 770 thousand tons in 2023 (see Figure 1) [63], and this represents a market value of 1.48 USD billion in 2023 and is estimated to grow by 8.1% annually until 2031 [64]. This amino acid can be produced by chemical synthesis and protein hydrolysis using enzymes. However, these alternatives are not cost‐benefit effective, which makes microbial fermentation the most efficient, commonly used way [65], and the microorganisms used the most are *Ec* and *Corynebacterium glutamicum (Cg)* since the genetics of both species are widely known, and their easy manipulation, study and scope as a molecular model [66]. However, production yield through *Cg* is usually lower than with *Ec*, which could be due to thicker cell walls in *Cg* than in *Ec* [44]. Thr production through bacterial fermentation using *Ec* is an efficient, safe and convenient method for the user [67].

## 7. L‐Threonine Production Strategies and Optimizations

**Figure 2:**
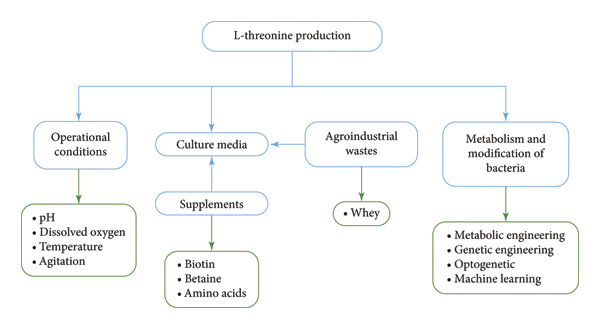
Summary of strategies for increasing and/or improving Thr production through microbes.

Thr production through bacterial fermentation has been carried out for over 60 years. During this time, methods to improve yielding have been refined, which is possible by modifying operational variables such as temperature, pH and oxygenation level (see Figure 2). It is also possible to change the culture medium to make it more nutritious and cheaper, supplement a product that can interact in metabolism as a potentiator or inhibitor, improve the culture qualities or help microorganisms to stay healthy; in cases of crossed‐contamination infection, and depending on the type of infection agent, antibiotics are used in the culture to eliminate invading microorganisms [73].

Some alternatives regarding culture mean modification, operational parameters and genetic modifications on strains to make them super productive have been explored lately, aiming to trigger a metabolic response that increases Thr production. When using whey as substrate, particular attention must be given to optimizing lactose metabolism and managing the complex nutrient profile. Among those, biotin supplementation in culture medium makes a fed batch and adjustments in oxygen supply, where the amount of oxygen available dissolved at a 20%, which is an important factor since high cellular density produced from a long fermentation process limits oxygen and increases accumulation of organic acids which, similarly, inhibit enzymes from carbon central metabolism, hindering its utilization and thus decreasing Thr production [50]. The use of acetate as a substitute carbon source has gained attention, as it can stimulate the tricarboxylic acid (TCA) pathway. Recent studies combining acetate utilization with metabolic engineering for homoserine and Thr production obtained the highest Thr values reported when applied to whey‐based media [74].

Betaine is an osmoprotective compound used as a supplement to improve Thr production, as it also positively regulates the *zwf* expression that codes for glucose‐6‐phosphate dehydrogenase, a key enzyme in glucose metabolism and whose activation results in a NAPH increase. Betaine can be taken through the osmotic transporter of H^+^ prop (coded by *prop*) or by the ABC‐type ProVWX transporter (coded by *proVWX*). Betaine is also produced by *Ec* itself because of *betAB1T*‐coded enzymes. The use of betaine has been combined with genetic engineering to develop strains with modification in the presence of *proP* and *ProVWX*, which caused an increase in Thr production due to the reduction in intracellular osmotic pressure [75].

As current research shows, metabolic engineering has become the primary approach to increasing Thr production (Table 4 shows relevant genetic modifications for Thr production carried out recently by *Ec*). Knowledge on metabolic flow has been used as the basis to implement methods such as feedback inhibition or repression by removing key enzymes or by regulating genes that code for the enzymes involved in Thr synthetic pathway. When working with whey as substrate, additional metabolic engineering considerations include optimization of lactose uptake and galactose metabolism pathways to maximize carbon flux towards Thr biosynthesis.

**Table 4:** Relevant genetic modifications for Thr production carried out recently by *Ec*.

Strain	Gene modification				Thr (g/L)	Reference
	Deletion	Insertion	Regulation	Overexpression		
W‐H31	*tdh*	*i* *l* *v* *A* ^*C*290*T*^		*aspA*, *ackA*, *acs*	45.80	[74]
TWF106, PST1011, pst1042pr		*lux* system	*ilvA*		118.20	[78]
THR36‐L19	*ilvA*, *tdh*, *crr*, *idhA*, *thrL*, *lacL*			*g* *l* *f*_*z* *m*_, *glk*, *t* *h* *r* *A* ^*G*433*R*^, *ppc*, *P* *y* *c*_*R* *e*_, *aspA*, *aspC*, *asd*/*a* *s* *d*_*t* *m*_, *thrB*, *thrC*, *rhtA*, *rhtC*, *pntAB*	120.00	[44]
TSW008, TSW009	*prop*, *betAB1T*, *proC*, *fadR*, *crr*, *ptsG*				24.90	[55]
TWF083		*P*_*c* *y* *s* *D*_, *P*_*c* *y* *s* *J*_, *P*_*c* *y* *s*_ *H*	*iCLR*, *aspC*, *arcA*, *cpxR*, *gadE*, *fadR*, *pykF*		116.62	[79]
ATC21277	*tdh*, *metL*, *dapA*, *dhaM*			*pntAB*, *ppc*, *aspC*	8.40	[47]
TWF044	*fadR*, *fabR*, *lacL*, *acs*	*aceBA*, fadBA, *P*_*tac*‐tre_			103.84	[49]
TH‐103Z, TH‐113Z, TH‐109Z			*ftsZ*		160.30	[75]
THR‐48, THR‐50			*fur*		154.20 (glucose) 92.46 (molasses)	[48]

New methods have been explored in the past 5 years that have shown excellent results, such as the creation of polyploid *Ec* that has 2–4 chromosomes using the *ftsZ* gene regulation, which codes for the cell division protein. This method was used to produce a strain with larger cellular size and resistance to a lower pH, producing up to 160 g/L of Thr [76]. Optogenetics has also been explored to regulate the bacterial fermentation process, thus discovering the inhibitory effect of blue light on *Ec* cellular signalling. This method, along with the creation of a high‐efficiency biofilm formation system, improved production from 10.12 to 16.57 g/L of Thr in a period of just 6 h. Optogenetics, biofilm formation and the modification of the cellular division mechanism are promising fields that just started to be approached and require extensive research [77].

**Figure 3:**
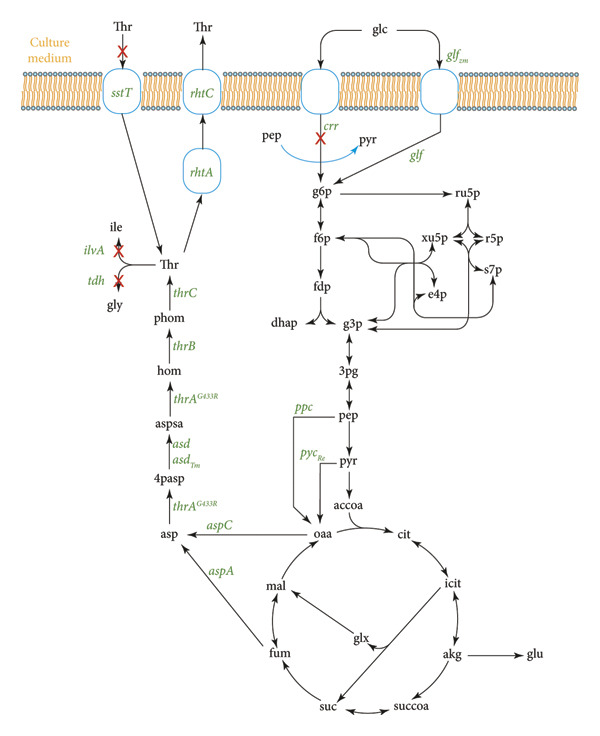
Schematic of Thr synthesis in engineered *Ec*. Adapted from [44].

One of the most current and promising strategies to increase Thr biosynthesis is multimodule metabolic engineering, where the metabolic pathway is divided into key modules including the Thr synthesis pathway, the supply of precursors, the Thr transport system, the supply of cofactors and the uptake and utilization of carbon sources (see Figure 3). Broad strategies include overexpression of carbonic anhydrase to increase CO_2_ fixation, introduction of the nonoxidative glycolysis pathway to increase acetyl‐CoA synthesis and use of quorum‐sensing systems to dynamically regulate key enzymes [44].

### 7.1. Economic Feasibility and Production Costs

The economic analysis presented herein is conducted at pilot demonstration scale (10,000‐L bioreactors, approximately 33.8 tons/year) and emphasizes comparative cost evaluation between substrates rather than absolute market competitiveness. Pilot‐scale production costs naturally exceed industrial market prices (1.31–1.66 USD/kg for feed‐grade Thr) due to well‐documented economies of scale; industrial fermentation facilities operating at 50,000–100,000 tons/year typically achieve 50%–60% cost reduction compared to pilot scale [43]. The primary value of this analysis lies in demonstrating that whey maintains a consistent 7.4% comparative advantage over glucose at equivalent scale, an advantage expected to persist at industrial scale due to whey’s intrinsic cost benefit as a waste‐derived substrate.

A technoeconomic assessment based on a 10,000‐L industrial batch scenario (see Table 5) demonstrates that whey‐based production is economically viable and competitive. Following the cost estimation methodology proposed by Konzock and Nielsen [43], the analysis reveals a total production cost of 20.55 USD/kg for whey‐based L‐threonine, compared to 22.20 USD/kg for glucose‐based processes. Although standard glucose media typically achieve higher yields [44], the substantial savings in raw materials offset the efficiency gap. Specifically, utilizing whey as a low‐cost agroindustrial residue [35] eliminates the high acquisition costs associated with commercial glucose, positioning it as a strategic alternative substrate despite market volatility.

**Table 5:** Comparative production costs per batch (10,000 L) assuming a target titter of 75.1 g/L.

Cost component	Whey‐based (USD)	Glucose‐based (USD)	Difference
Raw materials (substrate + salts)	8639	10,401	Substrate savings
Operational costs (energy/labour)	2366	2215	+6.8%
Downstream processing	4431	4055	+9.3%
Total cost/batch	15,435	16,671	−7.4%

Estimated cost (kg)	**20.55**	**22.20**	Cost Advantage

*Note:* Source: Authors’ estimation based on 2025 market prices [80] and production parameters [43, 44]. The values for estimated cost are in USD/kg. This correction should be entered in the corresponding cell. We apologize for this error. The values shown for the Estimated Cost of 20.55 USD/kg and 22.20 USD/kg when using a whey‐based or glucose‐based culture media, respectively, represent the cost of producing 1 kg of Thr, taking into account raw materials, operational costs, and downstream processing.

Beyond direct cost comparison, whey valorization provides substantial strategic value through environmental and circular economy benefits: elimination of dairy industry disposal costs (typically 0.01–0.05 USD/kg paid for whey treatment), conversion of 190–200 million tons of annual global waste into valuable biochemicals, reduction of water pollution from cheese manufacturing and alignment with sustainability regulations that increasingly favour waste‐to‐product bioprocesses. These environmental benefits, combined with the demonstrated 7.4% comparative cost advantage, position whey‐based L‐threonine production as both technically feasible at pilot scale and strategically valuable for industrial biorefinery development.

### 7.2. DSP Challenges and Cost Implications

DSP represents a critical phase in the industrial production of Thr, typically accounting for 20%–40% of total production costs depending on the purification complexity [43]. While fermentation using whey offers significant substrate cost advantages, it introduces specific challenges in the recovery stages compared to defined glucose media. Whey‐based broths contain residual whey proteins, minerals and unconsumed lactose, which increase viscosity and fouling propensity in filtration membranes [81]. Consequently, our economic model estimates that DSP costs for whey are approximately 9.3% higher than for glucose‐based processes (4431 vs. 4055 USD/batch). This cost increase is primarily driven by the “Cell Separation” stage (Table 6), where whey requires more robust filtration systems to handle higher solid loads. However, standard purification steps such as ion‐exchange chromatography and crystallization show similar cost profiles for both substrates once the broth is clarified.

**Table 6:** Breakdown of downstream processing costs per batch (1000 kg/L‐threonine yield).

Processing step	Whey‐based (USD)	Glucose‐based (USD)	Impact of whey matrix
Cell separation/filtration	1051	876	Higher due to membrane fouling
Purification (chromatography)	1878	1878	Comparable resin usage
Crystallization	901	901	Standard operation
Drying and packaging	601	601	Standard operation

TOTAL DSP COST	**4431**	**4055**	**+9.3% Increase**

*Note:* The values for Total DSP Cost are in USD/batch. This correction should be entered in the corresponding cell. We apologize for this omission. The values shown for Total DSP Cost are 4,431 USD/batch and 4,055 USD/batch when using a whey‐based or glucose‐based culture media, respectively, and represent the combined costs of treating the culture media once each batch of Thr production is finished, in order to extract this amino acid from the culture medium and package it for sale. The processes taken into account include cell separation and clarification, membrane filtration, ion exchange chromatography, crystallization, and drying and packaging.

While the 9.3% DSP cost premium for whey‐based processing represents a real engineering challenge at pilot scale, this differential is substantially outweighed by the 17% raw material cost advantage, yielding a net 7.4% overall cost benefit for whey‐based production. At an industrial scale, dedicated process optimization—including advanced antifouling membranes, continuous chromatography systems and integrated waste treatment—can further narrow the DSP cost gap while maintaining substrate cost advantages. The technical feasibility of achieving required product purity (≥ 98.5% for feed grade, ≥ 99.5% for food grade) from whey‐based fermentation has been demonstrated; remaining optimization is primarily an engineering scale‐up question rather than a fundamental limitation.

## 8. Future Perspectives of Whey Utilization as a Culture Medium for L‐Threonine Production

Given the utilization of whey in bacterial fermentation [82] to generate different products, its feasibility in the production of amino acids such as Thr is well established [25]. In decades, this has shown a broad spectrum of biological, medical, pharmaceutical, cosmetic and nutritional applications that will continue to be expanded [83]. From a statistical perspective, its demand is expected to increase exponentially, reaching an estimated 69.11 USD billion by 2034 [84]. After reviewing the available information and conducting a comparative analysis with other agroindustrial substrates, whey emerges as a particularly strategic choice for sustainable amino acid production.

Colombia is one of the main whey producers in Latin America and, according to commercial projections [85, 86], its production will increase along with the growth in cheese consumption per capita. This represents an additional volume of whey that will require valorization strategies. The use of metabolic engineering and digital tools such as machine learning will be essential to ensure high Thr production without having to perform multiple costly trial and error tests [47, 87]. Additionally, the use of these tools makes it possible to predict which genes need modification to improve carbon flux from lactose and galactose, generating higher Thr yields from whey‐based media.

Scalability from laboratory to industrial scale presents both technical and economic challenges that must be systematically addressed. Laboratory‐scale fermentations (1–10 L) typically operate under highly controlled conditions with synthetic supplements and may not accurately reflect performance at larger scales. Pilot‐scale operations (100–1000 L) reveal critical issues including: (1) oxygen transfer limitations as culture volume increases, requiring optimization of aeration and agitation systems [88]; (2) heterogeneity in substrate composition between whey batches, necessitating robust online monitoring and control systems [89]; (3) thermal management challenges, as larger fermentation volumes generate more metabolic heat [90]; and (4) increased risk of contamination over longer processing times (24–72 h for typical Thr fermentation) [91].

Whey pretreatment requirements at an industrial scale differ substantially from laboratory procedures. While small‐scale operations can utilize laboratory filtration and batch autoclaving, industrial processes require continuous preprocessing systems. Key considerations include the following: (1) implementation of continuous or semicontinuous pasteurization systems (typically 72°C for 15 s) rather than batch autoclaving to preserve heat‐sensitive nutrients and reduce energy consumption; (2) multistage filtration including coarse filtration (100–200 μm) to remove particulates, microfiltration (0.2–0.45 μm) for microbial load reduction and potentially ultrafiltration for protein standardization; (3) in‐line pH adjustment and nutrient supplementation systems with real‐time monitoring; and (4) large‐scale storage with temperature control (4°C–8°C) and turnover management to ensure substrate freshness. Regarding economic feasibility, studies on whey biorefineries indicate that while capital investment for pretreatment infrastructure is significant, it typically represents 15%–20% of the total plant cost, a figure that is amortized by the reduction in waste management fees [92].

Bioreactor design for whey‐based fermentation must account for the substrate’s specific characteristics. Optimal designs incorporate: (1) Enhanced oxygen transfer capability through improved impeller design and increased aeration capacity, as lactose metabolism may have different oxygen demands than glucose; (2) robust foam control systems, as whey proteins can exacerbate foaming; (3) heat exchange capacity 15%–20% higher than glucose‐based processes due to higher metabolic heat generation from lactose metabolism; (4) sampling and monitoring ports for real‐time assessment of lactose consumption, biomass density and product formation; and (5) materials compatible with mildly acidic conditions (pH 6.0–7.0 typical for Thr fermentation) and resistant to protein fouling. Computational fluid dynamics modelling has proven valuable in optimizing mixing and mass transfer for large‐scale fermentation, allowing the simulation of gradients that are critical in industrial bioreactors > 10,000 L [93, 94].

Quality control and regulatory aspects present additional considerations for industrial implementation. Feed‐grade Thr production from whey requires compliance with feed safety regulations including the absence of pathogens, limits on heavy metals and mycotoxins, and verification of no antibiotic residues. Food and pharmaceutical‐grade products face more stringent requirements, including comprehensive impurity profiling, endotoxin testing and allergen declaration (whey is a known allergen source). Establishing robust quality management systems that ensure batch‐to‐batch consistency despite inherent variability in whey composition is critical. Implementation of process analytical technology frameworks with real‐time monitoring of critical process parameters and quality attributes can help maintain consistent product quality. Regulatory pathways for amino acids produced from waste‐derived substrates are well‐established in many jurisdictions, although documentation requirements may be more extensive than for synthesis from pure feedstocks [95–97].

The availability of up‐to‐date, synthesized information is fundamental to understanding the current scenario and the scientific and technological progress that will ground future research on this issue. Integration of artificial intelligence and advanced process control into whey‐based fermentation operations represents a promising frontier. Machine learning algorithms can predict optimal fermentation parameters based on real‐time substrate composition analysis, adjust feeding strategies dynamically to maximize yield and identify early indicators of contamination or process drift. Digital twin technology, creating virtual replicas of fermentation processes, enables rapid scenario testing and optimization without costly physical experimentation. These advanced tools are particularly valuable for whey‐based processes where substrate variability necessitates adaptive process control [78].

Even though there is significant progress in knowledge, there are some important gaps to fill, such as fermentation process optimization specifically for different whey types (acid vs. sweet, different cheese varieties), innovative technology requirements for product separation and purification that minimize environmental impact while maintaining economic viability and comprehensive life cycle assessments comparing whey‐based production across different amino acids and bioproducts. Future research should prioritize the following: (1) Development of robust *Ec* strains optimized for lactose and galactose utilization with enhanced tolerance to whey‐derived inhibitors; (2) integration of whey‐based amino acid production with broader dairy industry biorefinery concepts, potentially co‐producing multiple value‐added products; (3) exploration of continuous or semicontinuous fermentation modes to improve productivity and reduce batch‐to‐batch variability; (4) investigation of alternative downstream processing technologies such as simulated moving bed chromatography or membrane adsorption to reduce purification costs; and (5) comprehensive technoeconomic modelling at commercial scale incorporating real‐world substrate and energy costs to guide industrial decision‐making.

## 9. Conclusions

Whey, traditionally known as cheese production waste, has proven to be a by‐product with significant valorization potential in biotechnology. Its utilization as culture means to produce essential amino acids, such as L‐threonine, represents a sustainable solution to a large environmental issue, while it also responds to a growing global demand for L‐threonine for nutritional supplements and functional biomolecules.

This review has demonstrated through comprehensive comparative analysis that whey offers substantial technical and economic advantages over other agroindustrial substrates, including lower costs, favourable nutrient composition, minimal pretreatment requirements and significant sustainability benefits.

The integration of advanced metabolic engineering, optimized fermentation strategies and emerging technologies such as machine learning and optogenetics has enabled dramatic improvements in L‐threonine yields, with current production reaching competitive titters in engineered strains. Economic analysis reveals that whey‐based fermentation is cost‐effective compared to glucose‐based processes. While downstream processing costs are approximately 9.3% higher due to matrix complexity and purification requirements, this is effectively offset by the savings in substrate acquisition costs. Our technoeconomic assessment estimates production costs at 20.55 USD/kg compared to 22.20 USD/kg for glucose‐based processes (∼7.4% cost reduction), offering a viable business case when combined with waste management savings.

Downstream processing challenges, while real, are manageable through appropriate process design and optimization. The combination of enzymatic pretreatment, membrane filtration, ion‐exchange chromatography and crystallization can consistently achieve product purities > 99% from whey‐based fermentation broths. The additional downstream processing costs are economically justified by the competitive market price of whey compared to commercial glucose substrates.

Scalability analysis indicates that whey‐based L‐threonine production becomes increasingly favourable at larger scales, with pilot and industrial implementations demonstrating technical feasibility and robust process performance. Key success factors for industrial scale‐up include robust whey preprocessing systems, adaptive process control to manage substrate variability, optimized bioreactor designs for whey‐specific fermentation characteristics and integrated downstream processing addressing whey‐derived impurities.

From a sustainability perspective, whey valorization for L‐threonine production delivers multiple benefits: mitigation of whey disposal environmental impacts (3.5 kg BOD and 6.8 kg COD per 100 kg whey), reduction in carbon footprint by significantly lowering reliance on resource‐intensive crops, contribution to circular economy principles through waste‐to‐value transformation and potential for carbon credit generation providing additional economic value. These environmental advantages align with global trends towards sustainable manufacturing and may create market differentiation opportunities for whey‐derived amino acids.

The path forward for whey‐based L‐threonine production is clear: continued research and development in strain optimization, process integration and cost reduction will progressively strengthen its competitive position. The convergence of environmental imperatives, economic advantages and technical feasibility positions whey as a strategic biorefinery platform for sustainable amino acid production. Continuity in the research and development of this area is fundamental to fully realizing all its potential and achieving widespread industrial implementation.

**Nomenclature**
BOD: Biological oxygen demand
*Cg*
: Corynebacterium glutamicum
COD: Chemical oxygen demand
CSL: Corn steep liquor
DSP: Downstream processing
*Ec*
: *Escherichia coli*
Thr: L‐threonine

## Conflicts of Interest

The authors declare no conflicts of interest.

## Author Contributions

Sara Pineda Vélez: conceptualization; investigation; data curation; methodology; project administration; visualization; writing–original draft; and writing–review and editing. Yudy Natalia Cortés Velásquez: conceptualization; investigation; data curation; methodology; visualization; writing–original draft; and writing–review and editing. Claudia Patricia Sánchez Henao: conceptualization; funding acquisition; supervision; writing–review and editing; and project administration. Jhon Fredy Vélez Blandón: conceptualization; supervision; visualization; and writing–review and editing.

## Funding

No funding was received for this manuscript.

## Data Availability

The data that support the findings of this review are available in Zenodo at https://doi.org/10.5281/zenodo.18236860. These data were derived from the following resources available in the public [63, 65, 68–72].
